# Biofortification in Millets: A Sustainable Approach for Nutritional Security

**DOI:** 10.3389/fpls.2017.00029

**Published:** 2017-01-23

**Authors:** A. Vinoth, R. Ravindhran

**Affiliations:** T. A. Lourdusamy Unit for Plant Tissue Culture and Molecular Biology, Department of Plant Biology and Biotechnology, Loyola CollegeChennai, India

**Keywords:** millets, biofortification, macronutrients, micronutrients, nutritional security

## Abstract

Nutritional insecurity is a major threat to the world’s population that is highly dependent on cereals-based diet, deficient in micronutrients. Next to cereals, millets are the primary sources of energy in the semi-arid tropics and drought-prone regions of Asia and Africa. Millets are nutritionally superior as their grains contain high amount of proteins, essential amino acids, minerals, and vitamins. Biofortification of staple crops is proved to be an economically feasible approach to combat micronutrient malnutrition. HarvestPlus group realized the importance of millet biofortification and released conventionally bred high iron pearl millet in India to tackle iron deficiency. Molecular basis of waxy starch has been identified in foxtail millet, proso millet, and barnyard millet to facilitate their use in infant foods. With close genetic-relatedness to cereals, comparative genomics has helped in deciphering quantitative trait loci and genes linked to protein quality in finger millet. Recently, transgenic expression of zinc transporters resulted in the development of high grain zinc while transcriptomics revealed various calcium sensor genes involved in uptake, translocation, and accumulation of calcium in finger millet. Biofortification in millets is still limited by the presence of antinutrients like phytic acid, polyphenols, and tannins. RNA interference and genome editing tools [zinc finger nucleases (ZFNs), transcription activator-like effector nucleases (TALENs), and clustered regularly interspaced short palindromic repeats (CRISPR)] needs to be employed to reduce these antinutrients. In this review paper, we discuss the strategies to accelerate biofortification in millets by summarizing the opportunities and challenges to increase the bioavailability of macro and micronutrients.

## Introduction

Nutritional security is the key to improve the health status of the world’s population as mankind is primarily dependent on plant-based diets. Plants are the major source of nutrients essential for normal growth and development. However, half of the global population, especially people from Asia and Africa suffer from nutrition deficiency as they rely on cereal crops for food (White and Broadley, 2005; Hirschi, 2009; Zhao and McGrath, 2009). Biofortification is a food-based approach to overcome the nutrient starvation by delivering nutrient-dense crops at the door steps of poor populations (Bouis et al., 2011). Biofortification Challenge Program (BCP) under HarvestPlus-Consultative Group for International Agricultural Research (CGIAR) Micronutrients project has focused primarily on seven major staple crops (rice, beans, cassava, maize, sweet potato, pearl millet, and wheat) targeting three important micronutrients (Fe, Zn, and vitamin A) (Welch and Graham, 2004). In resource-poor countries of Asia and Africa, millets provide 75% of total calorie intake next to cereal grains with an average annual production of 14.2 and 12.4 million tons (Belton and Taylor, 2004; O’Kennedy et al., 2006). India is the leading producer of millets accounting for about 80% of the global millet production (Food and Agricultural Organization [FAO], 2015).

Millets are commonly referred as “small seeded grasses” which include pearl millet [*Pennisetum glaucum* (L.) R. Br.], finger millet [*Eleusine coracana* (L.) Gaertn], foxtail millet [*Setaria italica* (L.) Beauv], proso millet (*Panicum miliaceum* L.), barnyard millet (*Echinochloa* spp.), kodo millet (*Paspalum scrobiculatum*), and little millet (*Panicum sumatrense*). Among the millets, pearl millet occupies 95% of the production (Yadav et al., 2012; Yadav and Rai, 2013; Agricultural Statistics, Government of India, 2014; Nedumaran et al., 2014). Foxtail millet [*S. italica* (L.) P. Beauv] is the second largest crop among the millets, cultivated for food in semi-arid tropics of Asia and as forage in Europe, North America, Australia, and North Africa (Austin, 2006). Finger millet is the sixth largest crop under cultivation serving as the primary food for rural populations of East and Central Africa and southern India (Vijayakumari et al., 2003). Proso millet is a short-season crop cultivated in drier regions of Asia, Africa, Europe, Australia, and North America (Baltensperger, 2002; Kimata and Negishi, 2002). Barnyard millet is the fastest growing among the millets with a harvesting period of 6 weeks (Sood et al., 2015). It is predominantly cultivated in India, China, Japan, and Korea for food as well as fodder. Kodo millet is native to the tropical and sub-tropical regions of South America and domesticated in India 3,000 years ago (de Wet et al., 1983b). Little millet was domesticated in the Eastern Ghats of India occupying a major portion of diet amongst the tribal people and spread to Sri Lanka, Nepal, and Myanmar (de Wet et al., 1983a).

Millets are nutritionally superior to rice and wheat as they contain a high amount of proteins, dietary fibers, iron, zinc, calcium, phosphorus, potassium, vitamin B, and essential amino acids (Hegde et al., 2005; Saleh et al., 2013). But the presence of antinutrients like phytates, polyphenols, and tannins reduce the mineral bioavailability by chelating multivalent cations like Fe^2+^, Zn^2+^, Ca^2+^, Mg^2+^, and K^+^ (Oberleas, 1973; Gupta, 1980; Kumar and Chauhan, 1993; Abdalla et al., 1998; AbdelRahman et al., 2005). In addition, high amounts of protease and amylase inhibitors affect the digestibility of millet grains (Shivaraj and Pattabiraman, 1981; Pattabiraman, 1986; Joshi et al., 1999). The predominance of the antinutritional factors has thus rendered the orphan status to millets in terms of global economic importance.

Biofortified crops have been primarily developed through conventional breeding exploiting the natural genetic variation, with the exception of Golden rice (www.harvestplus.org). Millets exhibit vast genetic variability for key mineral elements like, iron, zinc, and calcium when compared to other cereal crops (Muthamilarasan and Prasad, 2015). Moreover, millets are drought tolerant crops (O’Kennedy et al., 2009), resistant to pests and diseases offering good insurance against crop failure in developing countries (Tsehaye et al., 2006; Reddy et al., 2011). In spite of the superior quality of millets, only pearl millet has been prioritized as crop of choice for iron biofortification in India. Therefore, vast potential exists to utilize the minor millets for biofortification. Biofortification in millets can be achieved through two strategies: (1) by enhancing the accumulation of nutrients in milled grains and (2) by reducing the antinutrients to increase the bioavailability of minerals. This review highlights the importance of germplasm characterization of millets to develop biofortified varieties and the use of omics approaches to enhance grain-nutrient density. Taking the leads from other cereal crops, we emphasize the application of genetic engineering and genome editing tools to facilitate nutrient accumulation in edible portions and to block the biosynthesis of antinutrients.

## Nutritional Significance

Millets are highly nutritious being rich source of proteins, vitamins, and minerals. About 80% of millet grains are used for food, while the rest is used as animal fodder and in brewing industry for alcoholic products (for detailed review, see Saleh et al., 2013; Shivran, 2016). The grains are ground into flour and consumed as cakes or porridges. In Asian countries, street food vendors serve less expensive, ready-to-eat millet-based foods for poor consumers. Millets are recommended for well-being of infants, lactating mothers, elderly, and convalescents. The grains release sugar slowly into the blood stream and thus considered “gluten-free” (Taylor and Emmambux, 2008). With high fiber and protein content, millets are preferred as dietary foods for people with diabetes and cardiovascular diseases (Muthamilarasan et al., 2016). In addition, they contain health promoting phenolic acids and flavonoids, that play a vital role in combating free-radical mediated oxidative stress and in lowering blood glucose levels (Hegde et al., 2005; Dykes and Rooney, 2006, 2007; Chandrasekara and Shahidi, 2010, 2011; Kim et al., 2011; Kunyanga et al., 2012). Pearl millet is rich in Fe, Zn, and lysine (17–65 mg/g of protein) compared to other millets (McDonough et al., 2000; Hadimani et al., 2001). Foxtail millet contains a high amount of protein (11%) and fat (4%). The protein fractions are represented by albumins and globulins (13%), prolamins (39.4%), and glutelins (9.9%). It is thus recommended as an ideal food for diabetics. It also contains significant amounts of potential antioxidants like phenols, phenolic acids, and carotenoids (Saleh et al., 2013; Zhang and Liu, 2015). Finger millet grains contain higher levels of minerals like Ca, Mg, and K (Saleh et al., 2013; Devi et al., 2014). Positive calcium content maintains healthy bones (Pettifor, 2004), while potassium prevents the onset of diabetes, renal and cardiovascular diseases (He and MacGregor, 2008). It also has high levels of amino acids like methionine, lysine and tryptophan (Bhatt et al., 2011), and polyphenols (Chandrasekara and Shahidi, 2011; Devi et al., 2014). Proso millet contains the highest amount of proteins (12.5%) while barnyard millet is the richest source of crude fiber (13.6%) and Fe (186 mg/kg dry matter) (Saleh et al., 2013). Barnyard millet grains possess other functional constituents’ viz. γ-amino butyric acid (GABA) and β-glucan, used as antioxidants and in reducing blood lipid levels (Kofuji et al., 2012; Sharma et al., 2016). With lowest carbohydrate content among the millets, barnyard millet is recommended as an ideal food for type II diabetics (Ugare et al., 2011). Kodo millet is bestowed with high magnesium content (1.1 g/kg dry matter). Millets are therefore consumed as multi-grains to reap the collective health benefits of nutrients.

## Characterization of Millet Germplasm for Grain Nutrients

**FIGURE 1:**
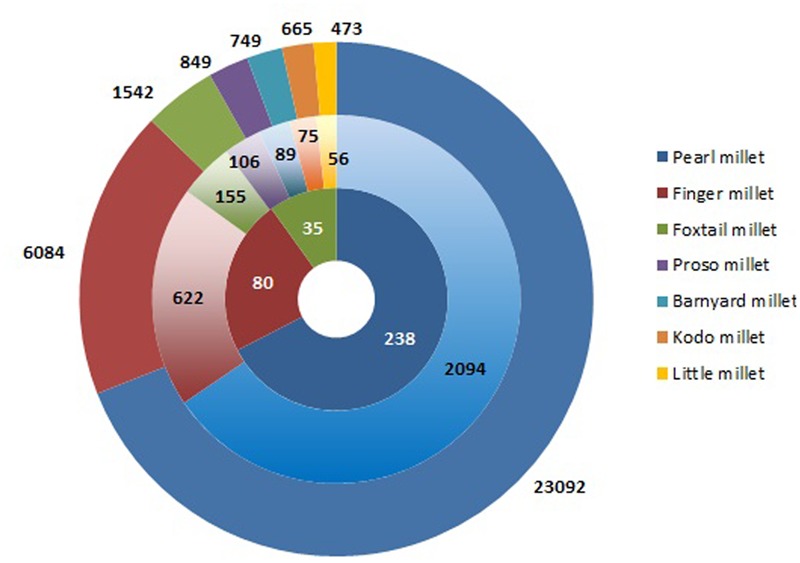
**Germplasm collection of millet accessions in ICRISAT genebank.** The outer concentric circle represents the entire collection of millets followed by reduced subsets of core and minicore collections in the inner circles.

Conservation of plant genetic resources (PGRs) provides a continuous supply of raw material for crop improvement. Success of biofortification program lies in the sustainable utilization of PGRs for nutritional enhancement (Muthamilarasan and Prasad, 2015). International Crop Research Institute for Semi-Arid Tropics (ICRISAT) contains the largest collection of millet germplasm representing 27.4% of total crop accessions in the genebank (**Figure 1**). Of this, pearl millet constitutes the vast majority of germplasm represented by 23,092 accessions including landraces, cultivars, genetic stocks, breeding lines, and wild relatives (Upadhyaya et al., 2016a). Finger millet germplasm consisting of 6,084 accessions is grouped under two subspecies, *africana* and *coracana* on the basis of morphology of inflorescence (Vetriventhan et al., 2016). Foxtail millet is a self-fertilizing species including 1,542 accessions from 23 different countries. Foxtail millet accessions are classified into three races, namely *indica, maxima*, and *moharia* and 10 subraces (Vetriventhan et al., 2016). Barnyard millet germplasm comprises of 749 accessions mainly from Japan and India (Upadhyaya et al., 2016c). The major collections of kodo millet from India and USA account for 665 accessions (Upadhyaya et al., 2016c). India is the prime contributor of little millet germplasm with 473 accessions (Upadhyaya et al., 2016c). Despite holding the largest millet germplasm, scientific community from India has made very few attempts to utilize the millet genomic resources for biofortification. This is mainly because of the scarcity of information on germplasm characterization for nutritional traits.

### Core and Minicore Collections

Characterization of entire germplasm for economically important traits is a daunting task for breeders. For the past two decades, germplasm characterization at ICRISAT has led to the establishment of core and minicore collections for pearl millet (Bhattacharjee et al., 2007; Upadhyaya et al., 2009, 2011a), finger millet (Upadhyaya et al., 2006), and foxtail millet (Upadhyaya et al., 2008, 2011b) while only core collections was established for other small millets (Upadhyaya et al., 2014) (**Figure 1**).

Trait-specific germplasm characterization is a prerequisite to identify genotypes contrasting for desirable traits. All India Coordinated Small Millets Improvement Project implemented by 1986 has focused mainly on varietal development for high yield and disease resistance with due negligence on nutritional quality (Shivran, 2016). Only by the start of the 21st century, millet germplasm received scientific attention for nutrition traits. Screening of pearl millet, foxtail millet, and finger millet accessions for grain nutrients revealed sufficient genetic variability. Multi-location on farm trials identified nutritionally superior lines with farmer preferred traits such as earliness to flowering and grain yield, adapted to local environments (Upadhyaya et al., 2011c; Muthamilarasan and Prasad, 2015). This process accelerates the pace of breeding in millets by studying the inheritance pattern and genotype–environment interaction for grain nutrients. Hybridization between diverse genotypes will generate mapping populations for DNA marker-assisted tagging of genomic regions linked to grain nutrients. However, molecular characterization of other minor millets for nutritional traits is limited and needs special attention. A thorough evaluation of small millets core collections for nutritional traits is the need of the hour to bring genetically diverse parents into mainstream breeding for generating recombinant inbred lines suitable for biofortification. The following section details the utilization of millet germplasm in biofortification in millets under the major headings: macronutrients, micronutrients, and antinutrients.

## Macronutrients

### Starch

Millets are the primary source of carbohydrates in tropics and semi-arid tropics of India and sub-Saharan Africa (Shivran, 2016). Grain starch typically comprises of two polymers, amylose (15–30%) and amylopectin (70–85%). Based on the amylose content, millet accessions are classified into two major phenotypes, waxy and non-waxy. Waxy grains containing 0% amylose and nearly 100% amylopectin are glutinous in nature, easily digestible and therefore recommended as food for infants under 6 years of age (Dreher et al., 1984; Englyst et al., 1992). Waxy mutants in staple crops have evolved during the domestication of landraces by human selection (Olsen et al., 2006; Fan et al., 2008). They have been identified in cereals and millets including rice (*Oryza sativa*; Isshiki et al., 1998), barley (*Hordeum vulgare*; Domon et al., 2002), sorghum (*Sorghum bicolor*; McIntyre et al., 2008), maize (*Zea mays*), foxtail millet (*S. italica*), proso millet (*P. miliaceum*), and barnyard millet (*Echinochloa* sp.) (Fukunaga et al., 2002a; Kawase et al., 2005; Kim et al., 2009). Amylose synthesis in millets is controlled by a single dominant waxy allele (*Wx*), while the recessive loss-of-function allele (*wx*) leads to the waxy phenotype with near 0% amylose content (Nakayama et al., 1998; Fukunaga et al., 2002a). In polyploid crops, mutations in different alleles of *Wx* loci produce low amylose, non-waxy and waxy phenotypes. Low amylose lines contain <3% amylose content due to the residual activity of non-mutant alleles (Fukunaga et al., 2002b; Graybosch and Baltensperger, 2009). Precise identification of mutations in low amylose and waxy mutants have led to the development of waxy starch foods.

**FIGURE 2:**
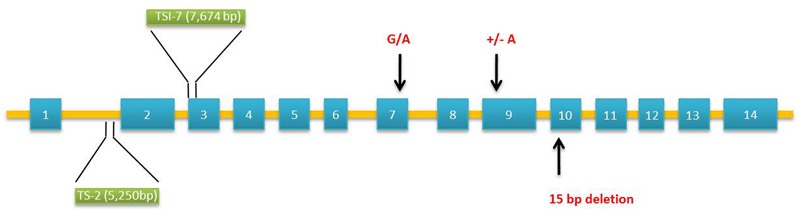
**Molecular structure of granule-bound starch synthase 1 (GBSS 1) gene in *Setaria italica* and *Panicum miliaceum* associated with waxy phenotypes.** Two polymorphisms occur in the *wx* allele of *Setaria*: insertion of transposable elements, TSI-2 (5,250 bp – Type IV) in intron 1 and TSI-7 (7,674 bp – Type V) in exon 3. Three SNPs occur in the exon sequence of waxy *Panicum*: 15-bp deletion [GCGCTGAACAAGGA GGCGCTG] in exon 10 (S type), insertion of an adenine residue in exon 9 leading to reading frameshift mutation (L type) and G/A substitution in exon 7 converting cysteine residue to tyrosine (L type). Exons and introns are denoted by boxes and bars, respectively.

The waxy gene product named as granule-bound starch synthase 1 (GBSS 1) is the key enzyme catalyzing the formation of amylose (Sano, 1984). Mutations in GBSS 1 result from insertions/deletions (InDels), transposable elements, and single base pair mutations (Isshiki et al., 1998; Domon et al., 2002; Van et al., 2008). In millets, *Wx* gene was found to contain 14 exons and 13 introns (**Figure 2**). Foxtail millet with waxy grains are cultivated in Japan, Taiwan, Philippines, and Indonesia (Takei and Sakamoto, 1989). Molecular analysis of foxtail millet waxy landraces by polymerase chain reaction (PCR) covering 4,006 bp of *Wx* gene using exon–intron primer sets (ex1/ex2 or ex2int1/ex4r) identified the insertion of transposable elements [transcriptionally silent information (TSI)-2 and TSI-7] in intron 1 or exon 3. All the waxy phenotypes harboring transposable elements in *Wx* loci differed in their geographical origin of domestication (Van et al., 2008). Next-generation sequencing technology has accelerated the re-sequencing of various crops to identify genome-wide nucleotide variations (Huang et al., 2009). High throughput analysis of genes and transcripts using genotyping by sequencing, transcriptome, and epigenetic analysis has been well documented as a powerful tool for identifying the genetic basis of nutritional traits in millets (Muthamilarasan and Prasad, 2015). The release of draft genome sequences of two foxtail millet cultivars, Yugu1 and Zhang gu has advanced the research for genetic improvement in foxtail millet (Bennetzen et al., 2012; Zhang et al., 2012). A foxtail millet waxy landrace, Shi-Li-Xiang (SLX) was re-sequenced using Solexa sequencing technology and Genome Analyzer II (GA II) to explore the nucleotide variations covering agronomic traits-related genes. Alignment with the reference genomes identified SNPs (single nucleotide polymorphisms), InDels, and SVs (structural variants). Novel markers obtained by re-sequencing helped in genome mapping of starch synthase encoding GBSS 1 peptide. Sequencing of GBSS 1 gene found the insertion of transposable elements confirming its waxy nature (Bai et al., 2013).

Waxy proso millet varieties are in existence since 1885 (Hixon and Brimhall, 1968). Graybosch and Baltensperger (2009) identified six waxy accessions of proso millet from the germplasm collection of United States Department of Agriculture-Agricultural Research Service (USDA-ARS) North Central Regional Plant Introduction (PI) Station, Ames, IA, USA. Based on the crosses between two (PI 436625 and PI 436626) waxy accessions and several wild type lines, two recessive alleles designated as *wx-1* and *wx-2* were found to be responsible for waxy phenotype. Grain starch amylose concentration was found to be reduced by 7.2-folds in waxy lines. However, a low level of amylose (3.5%) was present in some waxy types. This is most likely due to the low level of GBSS 1 activity produced by one of the alleles. The molecular basis of waxy endosperm in proso millet was further investigated using 38 landraces. Of these, 14 waxy phenotypes from China and Korea had little or no GBSS 1 activity. Sequencing of GBSS 1 in waxy lines revealed two different forms designated as “L type” and “S type” (**Figure 2**). These two forms differ in their exon sequence coding for mature GBSS 1 peptides resulting from three polymorphisms. The “S type” contains 15-bp deletion leading to the loss of five amino acids from *glucosyltransferase domain 1*. The “L type” contains a frame shift mutation caused by the insertion of an adenine residue and by substitution of a cysteine to tyrosine amino acid (Hunt et al., 2010). Based on these mutations, Araki et al. (2012) developed a PCR-based marker system (length polymorphism and derived cleaved amplified polymorphic sequences (dCAPS)) to identify waxy landraces amongst 83 accessions from Japan and 15 accessions from other countries. Sequencing of *wx* gene confirmed the ability of dCAPS markers in locating the single-bp mutations in exon 7 and 9 of waxy landraces.

Japanese barnyard millet is an allohexaploid and mutations in three alleles are considered to confer waxy nature. The probability of spontaneous mutations in all three alleles to obtain waxy cultivars is difficult. Wheat, also an allohexaploid was found to carry spontaneous mutations at one or two waxy loci (Nakamura et al., 1993a,b). Mutagenesis by chemical treatment on a low amylose wheat cultivar with non-functional *Wx-A1* and *Wx-B1* genes produced a waxy mutant (Yasui et al., 1997). With wheat as the model system, Hoshino et al. (2010) attempted to mutagenize barnyard millet landrace (Noge-Hie) into waxy phenotypes by treatment with Co^60^ gamma ray irradiation. One of the mutant plants showed significant reduction in amylose content. PCR analysis of waxy genes from this low amylose line using primers designed from the consensus sequences of foxtail millet, pearl millet, sorghum, and wheat detected the absence of a gene sequence specific for waxy phenotypes. Contrary to waxy wheat with more than one non-functional *Wx* allele, loss-of-function mutation in one allele was found to produce waxy cultivars in barnyard millet (Hoshino et al., 2010).

Traditional breeding in millets for waxy trait is a labor intensive and time consuming process. It took nearly 15 years to transform waxy trait into non-waxy elite foxtail millet cultivar Yugu1 through cross breeding (Quan et al., 2010). Marker-assisted selection accelerates the process of conventional breeding. But the information on molecular markers linked to waxy traits in millets lags far behind cereals. Henceforth, genotyping of diverse millet germplasm by high throughput re-sequencing will facilitate the development of new molecular markers to map the waxy trait. Molecular analysis of waxy gene in foxtail millet, proso millet, and barnyard millet has identified mutations in one or more alleles. With recent advancements in genome editing, application of programmable site-specific nucleases is a straightforward approach to induce genetic mutations in non-waxy elite cultivars for transforming them into waxy phenotypes. Thus genomics approaches will speed up the genetic improvement in millets in a cost effective manner to produce biofortified varieties.

### Proteins and Amino Acids

High quality proteins are essential for physical and mental well-being of humans, especially children (Heine et al., 1995; Tomé and Bos, 2007). Cereal proteins deficient in essential amino acids like methionine, lysine, and tryptophan lead to malnutrition in developing countries (Nirgude et al., 2014). Cereal proteins contain 1.5–2% lysine and 0.25–0.5% tryptophan while estimated average requirement is 5% and 1.1% for lysine and tryptophan (Young et al., 1998). Finger millet on the other hand is high in essential amino acids than cereals (Mbithi-Mwikya et al., 2000). It is therefore a suitable model system to elucidate the genetic control of protein quality. High lysine and tryptophan in finger millet is attributed to the transcriptional regulation of amino acid catabolism genes by *Opaque2* (*o2*), a basic leucine zipper (bZIP) transcription factor. *o2* modifiers (*Opm*) downregulate lysine ketoglutarate reductase dehydrogenase (Kemper et al., 1999) and upregulate aspartate kinase (Brennecke et al., 1996) resulting in free lysine and tryptophan in endosperm. Genetic differences in *Opm* alleles of finger millet germplasm remained largely uncharacterized until 2012. In the preliminary study, finger millet genotypes were evaluated for diversity in seed protein content using PCR-based markers. Random amplified polymorphic DNA (RAPD) and simple sequence repeat (SSR) profiles revealed few unique bands discriminating high and low protein genotypes. This study laid the foundation to select superior genotypes for use in traditional breeding to produce high quality proteins (Kumar et al., 2012).

Molecular characterization of *Opm* alleles using SSRs and SNPs can effectively identify quantitative trait loci (QTLs) influencing amino acid content (Goron and Raizada, 2015). Genic SSRs are powerful tools to link the genetic maps of related species and alleles of genic SSRs are associated with structural variations in the gene that affect transcription and translation of proteins (Kashi and Soller, 1999; Andersen and Lübberstedt, 2003). Nirgude et al. (2014) reported higher level of polymorphism in *Opm* genes using genic SSRs than RAPD, genomic SSRs and Cytochrome P_450_ markers as reported by Panwar et al. (2010b). Utilizing the functional potential of comparative genomics, high tryptophan finger millet genotypes were identified from global collection using genic SSRs for *Opm* genes derived from expressed sequence tag (EST) sequences of rice, maize, and sorghum. Association mapping of SSR loci found two QTLs for tryptophan and one QTL for protein content. Interestingly, a 220-bp allele of SSR locus OM5 marker designed from the 27-kDa γ-zein gene of *Opm* was present mostly in the high tryptophan-containing genotypes (Babu et al., 2014). This marker could be employed in marker-assisted breeding for introgression of *Opm* allele into high yielding cultivars. Fine mapping of *Opm* genes linked to QTLs could lead to genetic enhancement of seed protein quality in cereals and small millets. Recently, 16 prolamin encoding genes called setarins have been identified in foxtail millet using computational approaches (Muthamilarasan and Prasad, 2015). Sequence alignment of setarin genes with other millets and cereals revealed least homology indicating their uniqueness in improved protein quality. Quantitative RT-PCR analysis of setarin genes confirmed their role in seed protein accumulation by overexpression in developing spikes (Muthamilarasan and Prasad, 2015). Preliminary clues of such crop-specific genes for high protein could be useful in engineering other millets and cereals for protein enrichment.

## Micronutrients

### Iron (Fe)

Iron (Fe) deficiency in India report 79% of pre-school children and 56% of women to be anemic (Krishnaswamy, 2009). Fe supplementation program in India since 1970 failed to address the issue of iron deficiency (Anand et al., 2014). Recognizing biofortification as a feasible alternative for Fe delivery, HarvestPlus has developed high Fe pearl millet by conventional breeding (HarvestPlus, 2009). The first step in breeding crops for better nutrition is to evaluate the genetic diversity of available germplasm for target nutritional trait. ICRISAT, a member of HarvestPlus undertook the process of screening pearl millet germplasm for sources of high Fe density. Early reports revealed positive correlation of Fe and Zn grain content with no significant correlation on grain yield and seed size (Velu et al., 2007, 2011; Gupta et al., 2009; Govindaraj et al., 2013). This suggested increase in Zn grain content as an associated trait while breeding for high Fe pearl millet.

Prospects of breeding pearl millet for high Fe content began in the year 2004 with screening of germplasm accessions, seed parents, open pollinated varieties (OPV), improved populations, and population progenies. Progenies derived from AIMP 92901, a high-yielding OPV, exhibited large intra-population variability with highest levels of Fe (about 76 mg/kg) and Zn (about 65 mg/kg) than their parental control (Velu et al., 2007). All the improved progenies were derived from *iniadi* germplasm. *Iniadi* landraces from African subcontinent have farmer preferred agronomic traits like early maturing, large seed size, compact panicles, disease resistance, and drought tolerance (Andrews and Anand Kumar, 1996; Rai et al., 2008). Hence, *iniadi* germplasm was exploited for further hybridization experiments to breed for high Fe content. Velu et al. (2008) identified ICTP 8203, a commercial OPV (Rai et al., 1990) released in India by 1998 and still under cultivation to possess highest Fe and Zn content. By the year 2012, high Fe biofortified variety “ICTP 8203 Fe 10-2” developed by progeny testing was commercially released in India. ICTP 8203 Fe 10-2 had 9% more Fe (71 mg/kg) and 11% more grain yield than the parental control (Rai et al., 2013).

Taking advantage of cross-pollination in pearl millet, breeders are in continuous search for nutritionally elite varieties suited for local environments. Rai et al. (2014) screened seed parent progenies and restorer parent progenies for Fe and Zn variability using X-ray fluorescence spectroscopy. The mean Fe density of these progenies increased by 5–66% than the control cultivars. As *Iniadi* germplasm is a promising source for high grain Fe and Zn densities, Rai et al. (2015) and Upadhyaya et al. (2016b) evaluated germplasm accessions and landraces using the inductively coupled plasma atomic emission spectroscopy. Significant variability was observed for Fe (51–121 mg/kg) and Zn (46–87 mg/kg) with positive correlation. Novel sources for developing nutrient-rich pearl millet include IP 17521 of Gnali (106.9 mg/kg), IP 11523 of Idiyouwe (106.5 mg/kg) and IP 17518 of Gnali (79.1 mg/kg), IP 11535 of Iniadi (78.4 mg/kg) for iron (Fe) content and zinc (Zn) content, respectively. These germplasm accessions with greater nutrient density than the commercial cultivar ICTP 8203 are valuable sources for introgressing high Fe and Zn into elite breeding lines. Continuation of breeding program with planned crosses will help in identifying nutrient-dense parental lines suited for rapidly changing climatic conditions.

High Fe biofortified pearl millet provides twofolds higher iron than modern wheat varieties. This led to increase in iron absorption by 5–10% in around 35 million people consuming biofortified pearl millet (Cercamondi et al., 2013; Kodkany et al., 2013). Feeding trial by Haas et al. (2013) revealed that consumption of 232 g iron biofortified pearl millet flour/day resolved 65% more iron deficiency in Indian school children. Similarly, a randomized efficacy trial of iron-biofortified pearl millet was conducted for 6 months on secondary school children in Maharashtra to assess the improvement in Fe status. The study population included children with high risk of iron deficiency, low-iron diets, and regular consumption of pearl millet. Iron intake of children consuming biofortified pearl millet far exceeded the EAR within 4 months. There was a phenomenal increase in their serum ferritin and total body iron levels. The dose of iron delivered was comparable to that achieved by supplementation trials (Finklestein et al., 2015). Promising results from bioefficacy studies is the clear evidence for the use of biofortified millets as a sustainable intervention in high risk populations to overcome the iron deficiency.

### Zinc (Zn)

**FIGURE 3:**
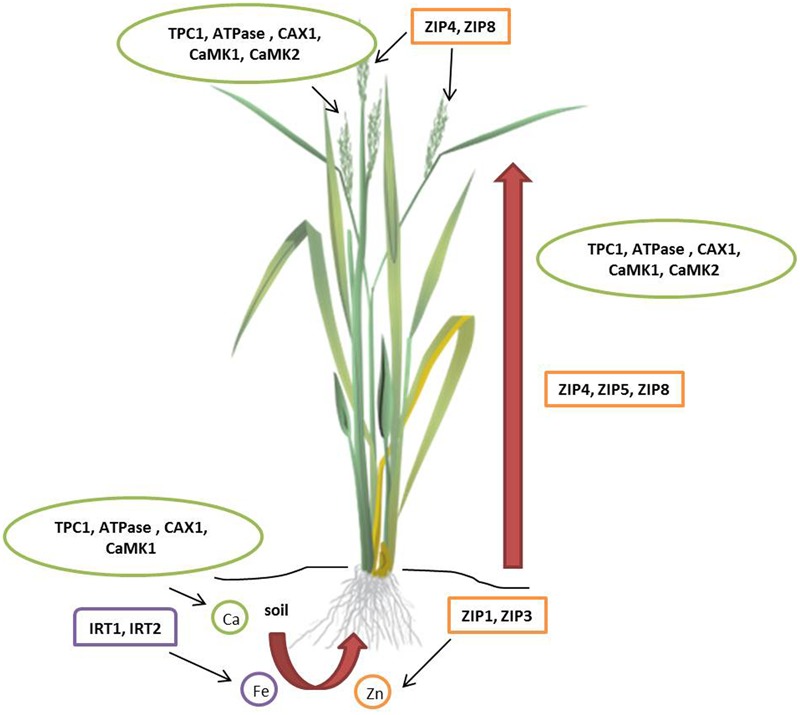
**Molecular targets for micronutrient accumulation in millets.** In rice (*Oryza sativa*), eight ZIPs facilitate iron and zinc uptake. *Os*ZIP1 and *Os*ZIP3 help in zinc uptake from soil; *Os*ZIP4, *Os*ZIP5, and *Os*ZIP8 translocate zinc from roots to shoots; *Os*ZIP4 and *Os*ZIP8 are involved in grain filling. Two Fe^2+^ transporters, *Os*IRT1 and *Os*IRT2 help in iron uptake from the soil. In finger millet (*Eleusine coracana*), calcium uptake involves three Ca^2+^ transporters and two calmodulin-dependent protein kinases (CaMKs). TPC1, ATPase, CAX1, CaMK1, and CaMK2 play a vital role in uptake, translocation, and accumulation of calcium. ATPase, CaM stimulated type IIB Ca^2+^ pump; CAX1, Ca^2+^/H^+^ antiporter or exchanger; TPC1, two-pore channel; ZIP, Zn-regulated transporters and Iron (Fe) regulated transporter-like protein.

Zinc (Zn) deficiency affects 50% of the world population resulting in diarrhea, impairment of physical growth, and suppressed immune function (Hotz and Brown, 2004; Gibson, 2006; Prasad, 2007; Qaim et al., 2007; Gibson et al., 2008). Breeding approach to improve the Zn grain content is discussed in the previous section. Genetic enhancement of grain Zn content is possible by modulating the metal transporters that facilitate their uptake, translocation, and storage. Members of Zn-regulated transporters and Iron (Fe) regulated transporter-like protein (ZIP) family contribute to Zn homeostasis by either uptake or remobilization in intracellular compartments (Guerinot, 2000; Mäser et al., 2001; Colangelo and Guerinot, 2006; **Figure 3**). ZIP transporters increase Zn uptake in several higher plants including *Arabidopsis thaliana* (Plaza et al., 2007), rice (*O. sativa*; Ishimaru et al., 2005; Bashir et al., 2012), barley (*H. vulgare*; Pedas et al., 2008), soybean (*Glycine max*; Moreau et al., 2002), and tomato (*Solanum lycopersicum*; Eckhardt et al., 2001). ZIP transporters are differentially regulated in various tissues under Zn deprivation and abundance in soils (Ramesh et al., 2003). Cereals and millets with high Zn seeds can be engineered by seed-specific expression of ZIP transporters. Initial success in transgenic development for seed Zn accumulation was recorded in rice (Ramesh et al., 2004). Recently, high zinc accumulating finger millet transgenic plants were produced by overexpression of *OsZIP1* driven by constitutive (*35S*) and endosperm-specific promoters (*Bx17*). Seeds of T_1_ transgenic plants showed 10–15 mg/kg higher Zn than wild type and the difference further increased to 20 mg/kg in T_2_ generation. Interestingly, higher Mn (5–10 mg/kg higher than wild type) accumulation was also recorded in the seeds of transgenic plants (Ramegowda et al., 2013). The *in planta* evidence of Zn accumulation in seeds by upregulation of ZIP transporters is the key to develop high Zn biofortified millets. As millets exhibit high synteny with cereals, expression of heterologous Zn transporters need further investigation to enhance grain Zn content.

### Calcium (Ca)

Calcium (Ca) deficiency resulting from high intake of cereals leads to osteoporosis in women (Ross et al., 2011). Plants absorb calcium ions from the soil solution and translocate to different organs via xylem transport predominantly controlled by transpiration pull (Grusak, 2002). Non-exposed structures like developing seeds exhibit negligible rate of transpiration and are low in calcium as they acquire minerals through phloem. In reproductive tissues, ion transporter proteins direct calcium transport (White and Broadley, 2003; White, 2005; Panwar et al., 2010a). Henceforth, elucidation of role of calcium transporters in plants favors the development of Ca biofortified cereals. Finger millet containing about 5–30 times higher Ca than wheat and rice serves as a model system to understand seed calcium accumulation. Singh et al. (2014) undertook transcriptomics approach to characterize calcium sensor gene family from the developing spikes of finger millet using Illumina paired-end sequencing methods. This study included characterization, identification, classification, phylogeny, and pathway analysis of calcium sensor genes of two genotypes, GP-1(low calcium) and GP-45 (high calcium). In total, 82 calcium sensor proteins identified in the transcriptome of finger millet spikes were grouped into 25-calmodulin (CaM) and calmodulin-like proteins (CaML), 9-CDPK-related protein kinases (CRK), 9-calcineurin B-like protein (CBL), 23-CBL interacting protein kinases (CIPK), and 14-Ca^2+^-dependent and CaM-independent protein kinases (CDPK) genes. Comparative phylogenetic analysis of calcium sensor gene family in finger millet identified 12 calcium sensor genes diverse from the rice orthologs. In addition, abundance of calcium sensor gene expression in GP-45 compared to GP-1 proved the polygenic nature of calcium accumulation. Quantitative real-time PCR analysis revealed higher expression of *EcCIPK2, EcCIPK9*, and *EcCIPK11* genes in GP-45 in response to external calcium supplied to the rhizosphere resulting in increased seed calcium content. In a similar study, Mirza et al. (2014) identified higher expression of Ca^2+^/H^+^ antiporter (CAX1), two pore channel (TPC1), CaM-stimulated type IIB Ca^2+^ ATPase and two CaM-dependent protein kinase (CaMK1 and CaMK2) genes in GP-45 resulting in greater uptake, translocation, and accumulation of calcium (**Figure 3**). Higher expression of CAX1 genes and CaM isoform in developing spikes strongly influence positive seed calcium content. Multigene engineering for co-expression of calcium sensor genes in cereal crops and small millets will play a significant role in the production of transgenic biofortified varieties.

### Vitamins

Vitamin A deficiency is a serious threat to well-being of children and pregnant women in developing countries (World Health Organization [WHO], 2011). Biofortification of staple crops such as sweet potato, cassava, and maize for high provitamin A by conventional breeding has achieved great success (Bouis et al., 2011). Millet collections on the other hand lack sufficient genetic variation for beta carotene content (Buerkert et al., 2001; Upadhyaya et al., 2011a). Hence traditional breeding for high provitamin A in millets is not feasible. In such cases, transgenic approach favors metabolic engineering of vitamin biosynthesis in plants. Golden rice is a notable example for genetic modification of provitamin A in grains (Paine et al., 2005). Regulatory issues concerning the genetically modified (GM) crops are being revisited with scientific evidences to facilitate their commercial production. Therefore, research on GM millets with enriched vitamins is gaining momentum.

## Antinutrients

Antinutrients like phytic acid, polyphenols, and tannins in millet grains greatly reduce the bioavailability of minerals. Phytic acid (myo-inositol-1,2,3,4,5,6-hexakisphosphate or IP_6_) the major form of phosphorus in seeds chelates the mineral cations in protein storage vacuoles (Raboy et al., 2000; Raboy, 2009). Bioefficacy studies on biofortified crops is a clear evidence for the antagonistic effect of IP_6_ on mineral absorption. Hama et al. (2012) carried out bioefficacy studies of biofortified and traditional pearl millet varieties of Africa subjected to abrasive decortication. Iron content of biofortified varieties (Tabi and GB8735) was 72 and 67 mg/kg dry matter, respectively. Though it corresponds to target biofortification levels, high phytate content reduced the iron bioavailability in these varieties. On the other hand, Zn content in biofortified varieties was 56 and 41 mg/kg dry matter, respectively. Low phytate to zinc ratio did not affect the zinc absorption to a greater level. Likewise, increased levels of polyphenols in biofortified iron pearl millet and black beans also reduced the Fe bioavailability (Tako et al., 2014, 2015). This study confirmed the negative impact of polyphenols on mineral absorption using *in vivo* chicken model and *in vitro* digestion/Caco2-cell model. Antinutrients are commonly removed by decortication, malting, fermentation, roasting, flaking, and grinding. However large-scale industrial methods for processing of millets to produce novel functional foods are not well developed as that for other cereal crops (Food and Agricultural Organization [FAO], 2012). Reduction in antinutrients during plant growth and development is therefore a promising strategy to improve the bioavailability of minerals from nutrient-rich millets.

Phytic acid is greatly reduced in low phytate (*lpa*) mutants of rice, wheat, and maize (Larson et al., 2000; Pilu et al., 2003; Guttieri et al., 2004; Kim et al., 2008). In most of the cases, *lpa* mutants had negative effects on crop yield and other agronomic traits. Henceforth, genetic engineering was considered as a safer alternative to generate *lpa* mutants (Feng and Yoshida, 2004). Genes controlling phytic acid biosynthetic pathway has been well characterized in major cereal crops (Raboy and Bowen, 2006). Three enzymes (MIPS, myo-inositol-3-phosphate synthase; MIK, myo-inositol-3-phosphate 5/6-kinase; IPK1, Inositol 1,3,4,5,6-pentakisphosphate 2-kinase) expressed in different levels of the biosynthetic pathway are the molecular targets for producing low phytate crops (Stevenson-Paulik et al., 2002; Shi et al., 2005; Sweetman et al., 2006; Kuwano et al., 2009). Successful silencing of IPK1 gene by RNAi technology produced low phytate rice with no significant effect on yield parameters (Ali et al., 2009). Recently, insertional mutagenesis of IPK1 gene by site-specific nucleases such as zinc finger nucleases (ZFNs) resulted in low phytate maize (Shukla et al., 2009). Rapidly emerging genome editing tools thus have enormous potential to develop biofortified millets by precise engineering of phytic acid biosynthetic pathway.

## Conclusion and Future Prospects

Millets are highly nutritious crops feeding poor populations in Asia and Africa. Scientific research to utilize the highly nutritious millet crops to combat micronutrient malnutrition is still meager. With good grain qualities and significant amounts of essential amino acids, minerals, and vitamins, bioavailability of nutrients need further improvement by reduction of antinutrients or by the use of novel promoters. Elucidation of role of various transporters in nutrient uptake, translocation, and storage could help in localizing the macro and micronutrients in edible parts of millets. Establishment of minicore collection of other millets will accelerate the molecular characterization of genetically diverse germplasm for new sources of variation in nutritional traits. Identification of molecular markers such as SNPs and InDels linked to nutritional traits will decipher the information on candidate genes controlling these traits. As millets exhibit cross-genera transferability, introgression of nutrient-linked genes into other cereals can become feasible by the use of molecular breeding or genetic engineering. Advent of next-generation sequencing platforms favors rapid sequencing of millet genome. Omics information on millets should advance more rapidly as cereal crops in order to enhance their utilization in the fight against micronutrient malnutrition. Thus integration of knowledge on genomics, transcriptomics, proteomics, and metabolomics could promote millets as model systems for advancements in biofortification of staple crops.

## Author Contributions

AV conceptualized and wrote the manuscript. RR edited the manuscript and critically revised the manuscript for publication.

## Conflict of Interest Statement

The authors declare that the research was conducted in the absence of any commercial or financial relationships that could be construed as a potential conflict of interest.

The reviewer [TB] and handling Editor declared their shared affiliation, and the handling Editor states that the process nevertheless met the standards of a fair and objective review.
